# CircVAMP3: A circRNA with a Role in Alveolar Rhabdomyosarcoma Cell Cycle Progression

**DOI:** 10.3390/genes12070985

**Published:** 2021-06-28

**Authors:** Francesca Rossi, Alvaro Centrón-Broco, Dario Dattilo, Gaia Di Timoteo, Marco Guarnacci, Alessio Colantoni, Manuel Beltran Nebot, Irene Bozzoni

**Affiliations:** 1 Department of Biology and Biotechnology Charles Darwin, Sapienza University of Rome, 00185 Rome, Italy; franc.rossi@uniroma1.it (F.R.); alvaro.centronbroco@uniroma1.it (A.C.-B.); dario.dattilo@uniroma1.it (D.D.); gaia.ditimoteo@uniroma1.it (G.D.T.); guarnaccimarco94@gmail.com (M.G.); manuel.beltrannebot@uniroma1.it (M.B.N.); 2 Center for Life Nano- & Neuro-Science, Fondazione Istituto Italiano di Tecnologia (IIT), 00161 Rome, Italy; alessio.colantoni@uniroma1.it

**Keywords:** circVAMP3, AKT, m^6^A, alveolar rhabdomyosarcoma, cell cycle progression

## Abstract

Circular RNAs (circRNAs), a class of covalently closed RNAs formed by a back-splicing reaction, have been involved in the regulation of diverse oncogenic processes. In this article we describe circVAMP3, a novel circular RNA overexpressed in RH4, a representative cell line of alveolar rhabdomyosarcoma. We demonstrated that circVAMP3 has a differential m^6^A pattern opposed to its linear counterpart, suggesting that the two isoforms can be differently regulated by such RNA modification. Moreover, we show how circVAMP3 depletion in alveolar rhabdomyosarcoma cells can impair cell cycle progression, through the alteration of the AKT-related pathways, pointing to this non-coding RNA as a novel regulator of the alveolar rhabdomyosarcoma progression and as a putative future therapeutic target.

## 1. Introduction

Circular RNAs (circRNAs) are covalently closed single-stranded RNA molecules originated from a particular splicing event, called back-splicing, in which a 5’ splice site of a precursor molecule is joined to an upstream 3’ splice site, producing the circularization of the intervening exons [1,2].

In recent years, circRNAs have stepped up as a large class of eukaryotic transcripts, often conserved among different species and whose expression is modulated during physiological and pathological processes [1,2,3,4,5].

CircRNAs regulate gene expression at different levels: they can act as miRNA sponges [1,6,7], as protein scaffolds [8,9], as templates for cap-independent translation [10,11,12] and as regulators of parental gene transcription [13].

CircRNA functions and the modulation of their expression have been studied in a variety of cellular processes, such as proliferation, differentiation, metabolic reprogramming, cancer onset and progression [14,15,16]. Recently, it has also been demonstrated that circRNA biogenesis and translation can be modulated by N6-methyladenosine (m^6^A) RNA modification [17,18].

Rhabdomyosarcoma (RMS) is the most common soft-tissue paediatric malignancy, accounting for about 5% of all cancers in children [19]. The disruption of the skeletal muscle differentiation programme of mesenchymal cells committed to a myogenic lineage is thought to be at the basis of their malignant transformation into RMS cells [20]. RMS tumour classification is generally based on its histological characteristics. Among the main RMS subtypes, the embryonal (ERMS) and the alveolar (ARMS) account for 60% and 20% of all RMS cases, respectively [21].

ERMS typically affects children in their first decade of life, while ARMS preferentially affects adolescents and is characterised by a more unfavourable prognosis [21]. The 3-year overall survival can reach 70–90% for low-risk RMS, while for the high-risk tumours it drops down to 25–30% [22].

The ERMS subtype is generally represented by the RD cell line, derived from a pelvic embryonal rhabdomyosarcoma mass in a seven-year-old female child [23]. On the other hand, RH4 cells are commonly used as a model system for ARMS and were derived from a lung metastasis of a seven-year-old female child [23].

In this work, we describe a circRNA, named circVAMP3, highly expressed in ARMS cells, with a putative role in regulating the cell cycle via the AKT survival pathway.

## 2. Materials and Methods

### 2.1. Cell Cultures and Transfections

Human primary myoblasts were derived from a skeletal muscle biopsy from a two-year-old male child (Telethon Biobank). They were cultured in DMEM high-glucose (Sigma-Aldrich, Saint Louis, MO, USA), 10% fetal bovine serum (FBS, Sigma-Aldrich), 2 mM l-glutamine (Sigma-Aldrich), 50 mg/ml insulin (Sigma-Aldrich), 25 ng/ml FGFb (Merck Millipore, Burlington, MA, USA), 1 ng/ml EGF (Corning, Corning, NY, USA) and 1% penicillin-streptomycin (Sigma-Aldrich).

Human ERMS RD cells and ARMS RH4 cells were cultured in DMEM high-glucose, 10% FBS, 2 mM L-glutamine and 1% penicillin-streptomycin.

Cells were transfected with 30 nM siRNAs (either Dharmacon, Lafayette, CO, USA, or Qiagen, Hilden, Germany) and either Dharmafect-1 Transfection Reagent (Dharmacon, Lafayette, CO, USA) or Lipofectamine RNAiMAX (Life Technologies, Carlsbad, CA, USA), according to the manufacturer’s instructions. For METTL3 knock-down, a mix of three siRNAs was used. The medium was replaced 24 h after the transfection and cells were harvested after another 24 h, unless differently specified. A list of the siRNAs used in this work is provided in Supplementary Table S1.

### 2.2. RNA Isolation and RNase-R Treatment

Total RNA was extracted with Direct-zol RNA Miniprep kit (Zymo Research, Irvine, CA, USA) with a 15-minute DNase-I treatment according to the manufacturer’s protocol, or through a standard phenol-chloroform extraction protocol.

Reverse transcription reaction for routine experiments was performed using PrimeScript RT Reagent Kit (Takara Bio, Kusatsu, Japan), while for RNA derived from immunoprecipitation experiments the SuperScript VILO cDNA synthesis kit (Thermo Fisher Scientific, Waltham, MA, USA) was used, according to the manufacturer’s protocol.

qRT-PCR was performed using Life-Tech SYBR (Life Technologies) according to the manufacturer’s protocol. In routine experiments, RNA levels are relative to Gapdh mRNA and relative RNA quantity was calculated as the Fold Change (2^−∆∆Ct^) with respect to the control sample set as 1, unless differently specified.

RT-PCR was performed using MyTaq-HS DNA Polymerase (Meridian Bioscience, Cincinnati, OH, USA) according to the manufacturer’s protocol. To prepare the PCR amplicon for Sanger sequencing, the DNA band corresponding to the specific amplicon to be analysed was extracted from the agarose gel through the NucleoSpin Gel and PCR Clean-Up Kit (Macherey-Nagel, Düren, Germany), according to the manufacturer’s instructions.

A list of the oligonucleotides used in this work is provided in Supplementary Table S1.

RNase R (Lucigen, Middleton, WI, USA) treatment was performed as described in [10]. Then, an equal volume of RNase R-untreated or RNase R-treated RNA was reverse-transcribed and analyzed by qRT-PCR.

### 2.3. Nucleus-Cytoplasm Subcellular Fractionation

Nucleus-cytoplasm subcellular fractionation was performed using the Paris Kit (Thermo Fisher Scientific), according to the manufacturer’s instruction. RNA was extracted as previously described. For the reverse transcription reaction, an equal volume of cytoplasmic and nuclear RNA was used. For qRT-PCR data representation, ∆Cts were calculated as average nuclear Ct − average cytoplasmic Ct; nuclear RNA quantity was calculated as 2^−∆Ct^ and then converted into percentage.

### 2.4. Western Blot

Protein extraction and Western blot analysis were performed as described in [24]. The following primary antibodies were used for hybridization: anti-AKT (9272, Cell Signaling Technology, Danvers, MA, USA), anti-phospho-AKT Ser473 (9271, Cell Signaling Technology), anti-β-ACTIN (ACTB, A3854, Sigma-Aldrich), anti-β-CATENIN (A302-012A, Bethyl, Montgomery, TX, USA), anti-ERK1 (A302-060A, Bethyl), anti-METTL3 (ab195352, Abcam, Cambridge, UK), anti-YTHDC1 (ab122340, Abcam). Images were acquired using a ChemiDoc MP Imager (Bio-Rad, Hercules, CA, USA) and analysed using Image Lab 5.2.1 software (Bio-Rad). Whole images were adjusted in contrast and brightness when necessary. Protein samples were run twice to allow multiple hybridization for proteins with the same molecular weight. ACTB hybridization was performed for each running, but only one ACTB hybridization is shown. However, for protein quantifications each specific signal was compared with the corresponding ACTB hybridization.

### 2.5. M^6^A CLIP

M^6^A CLIP was performed according to the protocol described in [25] with some modifications. Briefly, total RNA was purified from RH4 cells; 20 μg of RNA was diluted in IP buffer supplemented with RNase Inhibitor (Thermo Fisher Scientific) and incubated with 5 μg of anti-m^6^A antibody (Abcam, ab151230) for 2 h at 4 °C rotating head-over-tail and crosslinked; 10% of the solution was saved to be used as input, the leftover incubated with protein A/protein G Dynabeads (Thermo Fisher Scientific) for 2 h at 4 °C. Bead-bound antibody-RNA complexes were washed and recovered. After phenol-chloroform extraction and precipitation, RNA was resuspended in 30 μL, and 7 μL were reverse-transcribed with VILO Superscript (Thermo Fisher Scientific) in a 10 μL reaction. qRT-PCR was performed to evaluate targets enrichment through a relative standard curve.

### 2.6. Flow Cytometric Analysis of Cell Cycle

Cells were fixed in 2 mL ice-cold 70% ethanol per 1 × 10^6^ cells and incubated at 4 °C overnight. Then cells were centrifuged for 5 minutes at 1000 rpm. Pelleted cells were resuspended in 100 μL PBS (Sigma-Aldrich) supplemented with 10 μL of 1 mg/mL RNase-A (Sigma-Aldrich), and then incubated at 37 °C for 30 minutes. 10 μL of 1 mg/mL propidium iodide (Sigma-Aldrich) was added, and cells were incubated in the dark for 5 minutes at RT. Samples were processed using a BD FACSCalibur Flow Cytometer (BD Biosciences, Franklin Lakes, NJ, USA) machine and Cell Quest Pro (BD Biosciences) software. Results were analyzed using ModFit 3.1 software (BD Biosciences).

### 2.7. RNA-Sequencing and Bioinformatic Analyses

Three biological replicates of RH4 cells in control conditions (si-SCR) and two biological replicates of RH4 cells upon circVAMP3 knock-down (si-circVAMP3) were collected for RNA-sequencing. Total RNA was extracted as described before and treated with DNase according to the manufacturer’s protocol.

TruSeq Stranded mRNA kit was used for cDNA library preparation. The sequencing reactions, performed on an Illumina NovaSeq 6000 Sequencing system at IIT-Istituto Italiano di Tecnologia (Genova, Italy), produced an average of 61.5 million 151-nucleotide-long paired-end reads per sample.

Due to a laser intensity drop in the last cycles of the sequencing reaction, the last 20 based of reverse reads exhibited low Phred quality scores. Trimmomatic software [26] was employed to cut reverse reads down to 130 nucleotides; a second Trimmomatic run, with parameters *ILLUMINACLIP:path/to/adapter:2:30:10:8:true MINLEN:35*, allowed to remove adapter sequences. Alignment to human GRCh38 genome and Ensembl 99 transcriptome [27] was performed using STAR aligner version 2.5.2b [28] with parameters *--outSAMstrandField intronMotif --outFilterIntronMotifs RemoveNoncanonical --outSAMattrIHstart 0 --outSAMtype BAM SortedByCoordinate --outFilterType BySJout --outFilterMultimapNmax 20 --alignSJoverhangMin 8 --alignSJDBoverhangMin 1 --outFilterMismatchNmax 999 --outFilterMismatchNoverLmax 0.04*. Reads mapping to each gene were counted using htseq-count software [29] with parameters *-m intersection-strict -f bam -s reverse*. Read count files were combined into a count matrix file, which was given as input to edgeR R package [30] for differential expression analysis, after removing genes with a CPM value (Counts Per Million) less than one in at least three samples. Model fitting and testing were performed using glmFIT and glmLRT functions. Gene-level FPKM values were calculated using *rpkm* function from the edgeR package. FDR and absolute log_2_ Fold Change cutoff for selecting significant differentially expressed genes were set to 0.05 and 0.58, respectively.

Data from differential gene expression analysis are provided in Supplementary Table S2.

To identify the circRNAs from RNA-sequencing data (GEO: GSE117609), reads were aligned to the human reference genome (GRCh38) using bwa-mem [31] with *-T 19* option. CircRNA detection in each sample was then carried out using CIRI2 [32].

## 3. Results

### 3.1. CircVAMP3 Is Highly Expressed in RH4 Cells

**Figure 1:**
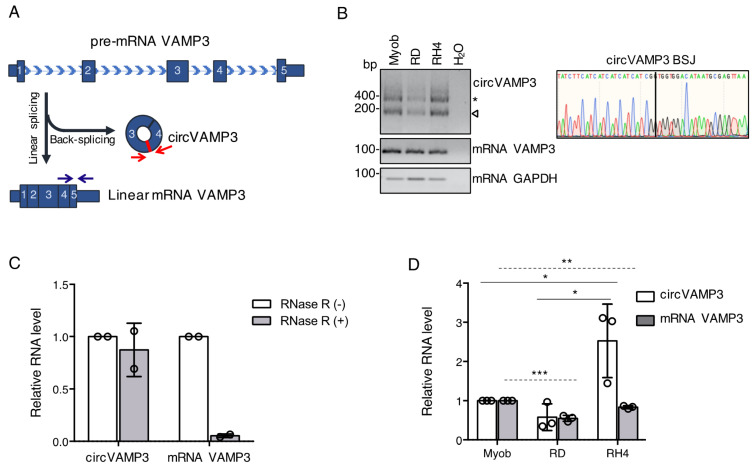
(**A**) Cartoon depicting the differential splicing of VAMP3 precursor transcript (pre-mRNA VAMP3) when it undergoes linear splicing to generate VAMP3 mRNA or back-splicing to produce circVAMP3. Convergent oligonucleotides to detect the linear mRNA and divergent oligonucleotides to detect the circRNA are depicted as arrows. Back-splicing junction (BSJ) is highlighted in red on circVAMP3 cartoon. (**B**) Left panel: RNA species detected by RT-PCR using oligonucleotides to detect circVAMP3, VAMP3 mRNA and GAPDH mRNA in myoblasts (Myob), RD, RH4 cells, and H_2_O negative control. CircVAMP3 oligonucleotides can amplify the circular RNA isoform (the band indicated by an arrow) and concatemers produced by consecutive reverse-transcription (the band indicated by an asterisk). Amplicon sizes (bp) are indicated on the left. Right panel: detail from the Sanger sequencing of circVAMP3 RT-PCR amplification, depicting the BSJ. (**C**) Relative RNA levels detected by qRT-PCR of circVAMP3 and VAMP3 mRNA after RNase R treatment. Data are represented as mean of fold changes ± standard deviation of two biological replicates, with dots representing individual datapoints. (**D**) Relative RNA levels of circVAMP3 and VAMP3 mRNA in myoblasts (Myob), RD and RH4 cells, detected by qRT-PCR. Data are represented as mean of fold changes ± standard deviation of three biological replicates, with dots representing individual datapoints. Continue horizontal bars refer to statistical confrontations for circVAMP3; dashed horizontal bars refer to statistical confrontations for linear VAMP3. Where statistical analysis was performed, the ratio of each sample versus its experimental control was tested by a two-tailed unpaired Student’s *t*-test. *: *p*-value < 0.05, **: *p*-value < 0.01, ***: *p*-value < 0.001, ****: *p*-value < 0.0001.

Vesicle-Associated Membrane Protein 3 (VAMP3) is a gene able to generate a protein belonging to the vesicle-associated membrane protein (VAMP)/synaptobrevin family. These proteins have an important role in vesicle-mediated transport and phagosome formation [33]. The VAMP3 *locus* is also able to generate a circular transcript corresponding to the circular RNA hsa_circ_0006354 (circVAMP3) [4,34,35]. This circRNA is produced by the back-splicing of the exons 3 and 4, generating a 211-nt covalently closed RNA molecule (Figure 1A).

From the analysis of RNA-sequencing data produced in our laboratory (GEO: GSE117609) [24], we detected both the circular and linear VAMP3 isoforms in human primary myoblasts, embryonal rhabdomyosarcoma (ERMS) RD cells and alveolar rhabdomyosarcoma (ARMS) RH4 cells. To validate their expression, we performed RT-PCR in all the three cell lines using specific divergent primers facing circVAMP3 back-splicing junction (BSJ) or specific convergent primers for VAMP3 linear mRNA (Figure 1A). The RT-PCR amplicon corresponding to the circular form (indicated by the arrow in Figure 1B, left panel) was purified and submitted to Sanger sequencing. The results of the sequencing confirmed the presence of the BSJ in the circVAMP3 amplicon, confirming the circularization of the RNA (Figure 1B, right panel). The slower migrating band (indicated by an asterisk in Figure 1B, left panel) was also sequenced and resulted to correspond to concatemers amplification likely originating by the reverse transcription of the circular molecule.

The covalently closed structure of circVAMP3 was confirmed by qRT-PCR on RNA from RMS cells either untreated (RNase R −) or treated with the RNase R exonuclease (RNase R +). As shown in Figure 1C, the addition of the exonuclease did not affect the levels of circVAMP3, whilst the signal detected in linear VAMP3 mRNA amplification dropped down upon the RNase R treatment. This demonstrates the covalently closed structure of circVAMP3 RNA in rhabdomyosarcoma cells.

We then performed a quantitative assay to evaluate changes in the expression of circVAMP3 and its linear counterpart among the three different cell lines (myoblasts, RD, and RH4 cells). As shown in Figure 1D, circVAMP3 was significantly more expressed in the RH4 ARMS cell line compared to myoblasts and RD ERMS cells, whilst the level of its linear counterpart slightly decreased, making particularly interesting the study of circVAMP3 in the RH4 cells.

### 3.2. CircVAMP3 Is Located in the Cytoplasm and Has a Differential m^6^A Methylation with Respect to Its Linear Counterpart

**Figure 2:**
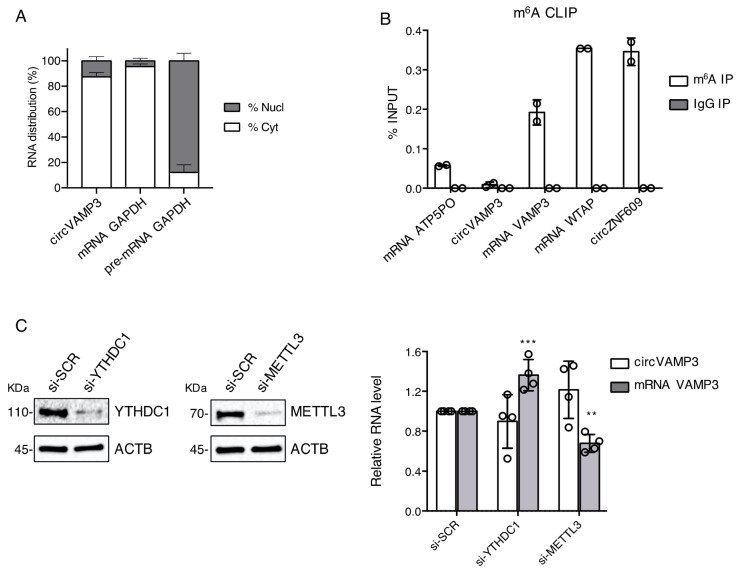
(**A**) Percentage of RNA distribution detected by qRT-PCR in the cytoplasmic (Cyt) and nuclear (Nucl) fractions of RH4 cells for circVAMP3, GAPDH mRNA (cytoplasmic localization control) and GAPDH pre-mRNA (nuclear localization control). Data are represented as mean of fold changes ± standard deviation of three biological replicates. (**B**) RNA levels relative to the input after immunoprecipitation using specific antibodies against either m^6^A RNA modification or IgG. Data are represented as mean ± standard deviation of two biological replicates, with dots representing individual datapoints. (**C**) Left panel: Western blot in RH4 cells in si-SCR, si-METTL3 and si-YTHDC1 conditions to detect YTHDC1, METTL3 and ACTB (loading control) protein levels; representative experiment from four independent replicates; protein molecular weights (KDa) are indicated on the left. Right panel: Relative RNA levels detected by qRT-PCR of circVAMP3 and VAMP3 mRNA in RH4 cells after transfection with either si-SCR, si-YTHDC1 or si-METTL3. Data are represented as mean of fold changes ± standard deviation of four biological replicates, with dots representing individual datapoints. Where statistical analysis was performed, the ratio of each sample versus its experimental control was tested by a two-tailed unpaired Student’s *t*-test. *: *p*-value < 0.05, **: *p*-value < 0.01, ***: *p*-value < 0.001, ****: *p*-value < 0.0001.

To better characterise circVAMP3 in RMS cells, we performed a cytoplasmic/nuclear fractionation in RH4 ARMS cells followed by qRT-PCR analysis in each fraction. As shown in Figure 2A, circVAMP3 is mainly localized in the cytoplasm similarly to GAPDH mRNA, used as the cytoplasmic localization control. Since previous work conducted in our laboratory demonstrated that m^6^A modification has an important role in circRNA metabolism [18], we checked for circVAMP3 m^6^A content in RH4 cells. M^6^A CLIP assay performed on total RNA from RH4 cells showed an enrichment only of the linear VAMP3 isoform (Figure 2B). The levels of the linear VAMP3 enrichment were higher than the negative control ATP5PO mRNA and almost comparable to that of the positive controls WTAP mRNA and circZNF609 [10,18]. To test if the differential methylation pattern has some implications in the regulation of the linear versus the circular VAMP3 isoforms, we performed a siRNA-mediated depletion of METTL3 and YTHDC1 (Figure 2C, left panels), a writer and a reader of the m^6^A modification respectively. As depicted in Figure 2C (right panel), their depletion did not alter the levels of circVAMP3 significantly. On the contrary, the interference of YTHDC1 produced a significant increase of the linear VAMP3 mRNA while the depletion of METTL3 led to its significant decrease. Our data indicate a clear difference in the methylation status between the circular and the linear isoforms making only the linear mRNA sensitive to the m^6^A-mediated regulation.

### 3.3. CircVAMP3 Knock-Down Affects Proliferation-Related Pathways in ARMS Cells

**Figure 3:**
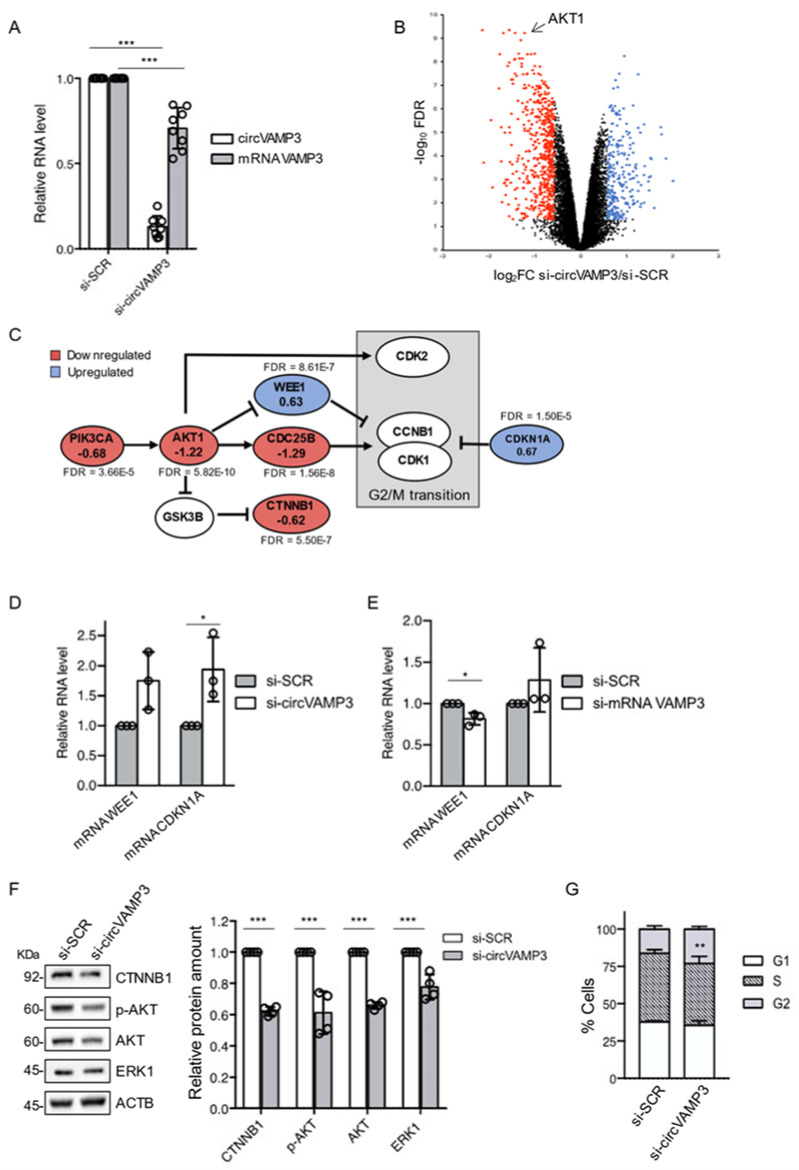
(**A**) Relative levels of RNA detected by qRT-PCR of circVAMP3 and VAMP3 mRNA in RH4 cells treated with si-SCR or si-circVAMP3. Data are represented as mean of fold changes ± standard deviation of 8 biological replicates, with dots representing individual datapoints. (**B**) Volcano plot showing genes differentially expressed in RH4 cells treated with si-circVAMP3 vs si-SCR. In blue, genes with a log_2_ fold change (FC) greater than 0.58 and an FDR lower than 0.05. In red, genes with a log_2_FC lower than −0.58 and an FDR lower than 0.05. AKT1 gene is indicated by an arrow. (**C**) Cartoon depicting the relation of several proteins belonging to the AKT pathway and its relationship with the G2/M transition pathway; proteins downregulated at the RNA level upon circVAMP3 knock-down are coloured in red, while the upregulated ones are coloured in blue. The numbers in the circle represent the log_2_FC, and the FDR is indicated below each circle. (**D**,**E**) Relative levels of RNA detected by qRT-PCR of WEE1 and CDKN1A transcripts in RH4 cells treated with si-SCR and either si-circVAMP3 (**D**) or si-mRNA VAMP3 (**E**). Data are represented as mean of fold changes ± standard deviation of three biological replicates, with dots representing individual datapoints. (**F**) Left panel: Western blot in RH4 cells treated either with si-SCR or si-circVAMP3 to detect CTNNB1, phosphorylated-AKT (p-AKT), AKT, ERK1 and ACTB (loading control) protein levels. Protein molecular weights (KDa) are indicated on the left; the panel shows a representative experiment of four. Right panel: Quantification of protein levels (relative to ACTB) from the experiment shown in the left panel. Data are represented as mean of relative protein levels ± standard deviation of four biological replicates, with dots representing individual datapoints. (**G**) Cell cycle analysis by flow cytometry (FACS) of RH4 cells upon control treatment (si-SCR) or circVAMP3 knock-down (si-circVAMP3). Data are represented as mean % cells in each cell cycle phase ± standard deviation of four biological replicates. Where statistical analysis was performed, the ratio of each sample versus its experimental control was tested by a two-tailed unpaired Student’s *t*-test. *: *p*-value < 0.05, **: *p*-value < 0.01, ***: *p*-value < 0.001, ****: *p*-value < 0.0001.

In order to study the possible role of circVAMP3 in RH4 cells, we designed a siRNA against its BSJ (Supplementary Figure S1A), which is the unique sequence distinguishing the circRNA from its linear counterpart. We treated RH4 ARMS cells with either control si-SCR or si-circVAMP3 and, as can be observed in Figure 3A, si-circVAMP3 produced a strong downregulation (87%) of circVAMP3 when compared to the control si-SCR condition, with a little but still significant downregulation of its linear counterpart (30%).

We then subjected samples from RH4 cells either treated with si-SCR or si-circVAMP3 to poly-A^+^ RNA-sequencing, to identify transcripts with a significant differential expression between the two conditions (Figure 3B, Supplementary Table S2).

Among the targets with the most significant downregulation, we identified AKT1 (Figure 3B, arrow). Besides AKT1 we also found AKT-pathway related genes to be significantly deregulated (Figure 3C). These genes are master regulators of several aspects of cell fate, including proliferation, survival as well as malignant transformation. Growth-factor-mediated activation of the AKT signalling induces normal cell cycle progression by acting on diverse downstream factors controlling the G1/S and G2/M transitions [36]. Among the factors involved in the AKT pathway, we could detect a significative downregulation of the cell-cycle promoters PIK3CA and CDC25B in si-circVAMP3 treated cells, and an upregulation of cell-cycle inhibitors such as WEE1 (Figure 3C). Moreover, other important regulators of the cell cycle linked to the AKT pathway were altered in si-circVAMP3 cells such as the β-Catenin (CTNNB1) and the cyclin inhibitor CDKN1A (P21), down- and up-regulated respectively (Figure 3C, Supplementary Table S2).

We then checked by qRT-PCR the RNA levels of the downstream inhibitors of the G2/M transition, the WEE1 and CDKN1A factors. Upon circVAMP3 knock-down we confirmed the RNA-sequencing results, observing an increase of both transcripts (Figure 3D), even though WEE1 did not reach statistical significance (*p*-value = 0.0532) due to variability among the experiments. Since the circVAMP3 knock-down produced a significant reduction in the VAMP3 mRNA levels (about 30%, Figure 3A), we checked the effects of the specific knock-down of VAMP3 mRNA in our system (Supplementary Figure S1B).

Figure 3E shows that the linear VAMP3 mRNA does not contribute to the upregulation of WEE1 and CDKN1A mRNAs; in fact, its knock-down produced a mild downregulation of the WEE1 transcript and no significant effects on CDKN1A mRNA (Figure 3E). These data indicate the specific effect of circVAMP3 on these two essential cell cycle regulators.

We then proceeded to the validation of other upstream factors related to the AKT pathway, such as β-Catenin (CTNNB1), phosphorylated-AKT (p-AKT), AKT and ERK1 proteins, in RH4 cells upon si-SCR or si-circVAMP3 treatment. The Western blot of Figure 3F shows that si-circVAMP3 produces a clear and significative downregulation of β-Catenin, phosphorylated and total AKT, and ERK1 proteins. As control, the downregulation of the linear VAMP3 isoform had no similar effects on the levels of AKT and ERK1 (Supplementary Figure S1C), indicating that the effect on these factors is specific for the circVAMP3 depletion. However, we noticed a downregulation of CTNNB1 and p-AKT proteins (Supplementary Figure S1C), indicating a contribution of the linear VAMP3 RNA in the regulation of these two specific proteins.

On the overall, the alteration of the homeostasis of such important regulators might play important roles in survival/proliferation pathways such as those involved in the G2/M transition.

To detect some downstream effects of the AKT pathway alteration produced by circVAMP3 depletion, we investigated whether si-circVAMP3 treatment might produce a change in RH4 cell cycle progression. Therefore, we performed a FACS cell cycle analysis using propidium iodide staining in RH4 cells treated either with si-SCR or si-circVAMP3. As shown in Figure 3G, circVAMP3 knock-down led to a small but significant increase in the percentage of cells in G2, suggesting a possible blockage at the early M-phase checkpoint.

## 4. Discussion and Conclusions

Rhabdomyosarcoma is the most common soft-tissue pediatric cancer, in which the alteration of the skeletal muscle differentiation programme is thought to drive the malignant transformation of mesenchymal cells committed to a myogenic lineage [20]. The discovery of putative novel biomarkers and therapeutic targets is paramount to improve the prognosis of the illness. CircRNAs have been demonstrated to play an important role in a variety of biological processes. They can regulate cell proliferation, metabolic reprogramming, angiogenesis, and metastasis [14,15,16], hence their description and classification are an important matter in the present research. 

Here we describe and characterize circVAMP3, a circRNA involved in cancer cell proliferation. Particularly, the knock-down of circVAMP3 impairs the cell cycle progression of RH4 cells, a representative cell line for alveolar rhabdomyosarcoma (ARMS), through the regulation, among others, of the AKT pathway. It is important to underline that the homeostasis of the circVAMP3 and its linear counterpart could be under a more complex regulation, since the knock-down of circVAMP3 also produces a slight downregulation of VAMP3 mRNA. However, it seems that the downregulation of circVAMP3, and not of its linear counterpart, is specifically responsible for the upregulation of CDKN1A, WEE1, which directly regulate the CCNB1/CDK1 complex controlling the G2/M checkpoint, as well as the downregulation of AKT and ERK1, therefore leading to an accumulation of cells in the G2 phase.

Although circVAMP3 and its linear counterpart are both expressed in myoblasts, RD and RH4 cells, they seem to be subjected to a different regulation in the three cell lines. Indeed, while the linear VAMP3 mRNA shows a downward trend between myoblasts and RMS subtypes, the circular RNA displays a strong upregulation only in RH4 cells. This suggests the existence of cell-specific mechanisms regulating circVAMP3 expression which differ from those that control the linear counterpart. The factors regulating the alternative biogenesis of circular and linear isoforms are still largely unknown; however, m^6^A modification has recently been demonstrated to participate in this choice [18] and to cooperate with factors regulating alternative splicing. The data here presented show that while the linear VAMP3 is methylated, circVAMP3 is not. These results unveil the existence of specific regulatory mechanisms controlling the alternative deposition of m^6^A modifications on the different transcripts originating from the same locus. The distinctive m^6^A signature will in turn impact on the function of the mature RNA species. Identifying the factors required to control linear and back-splicing for individual transcripts will also offer powerful tools to selectively target circRNA production for therapeutic purposes.

## Data Availability

RNA-sequencing raw data have been deposited at Gene Expression Omnibus (GEO) with the accession code GSE174397. This can be accessed with the secure token qnilgwwcxfejnit.

## Supplementary Materials

The following are available online at https://www.mdpi.com/article/10.3390/genes12070985/s1, Table S1: List of oligonucleotides and siRNAs used in this work. Table S2: List of differentially expressed genes in RH4 cells in control condition (si-SCR) or upon circVAMP3 knock-down (si-circVAMP3), RPKM = reads per kilobase per million reads. Figure S1: Supplementary Figure S1 providing details about circVAMP3 and VAMP3 mRNA knock-down.
