# Supplementary material for: CircVAMP3: A circRNA with a Role in Alveolar Rhabdomyosarcoma Cell Cycle Progression
Source: Genes (Basel). 2021 Jun 28;12(7):985. doi: 10.3390/genes12070985 (PMC8303801; doi:10.3390/genes12070985)
Supplement: Supplementary file 1 [file genes-12-00985-s001.zip › Figure S1.pdf]

Supplementary figure:

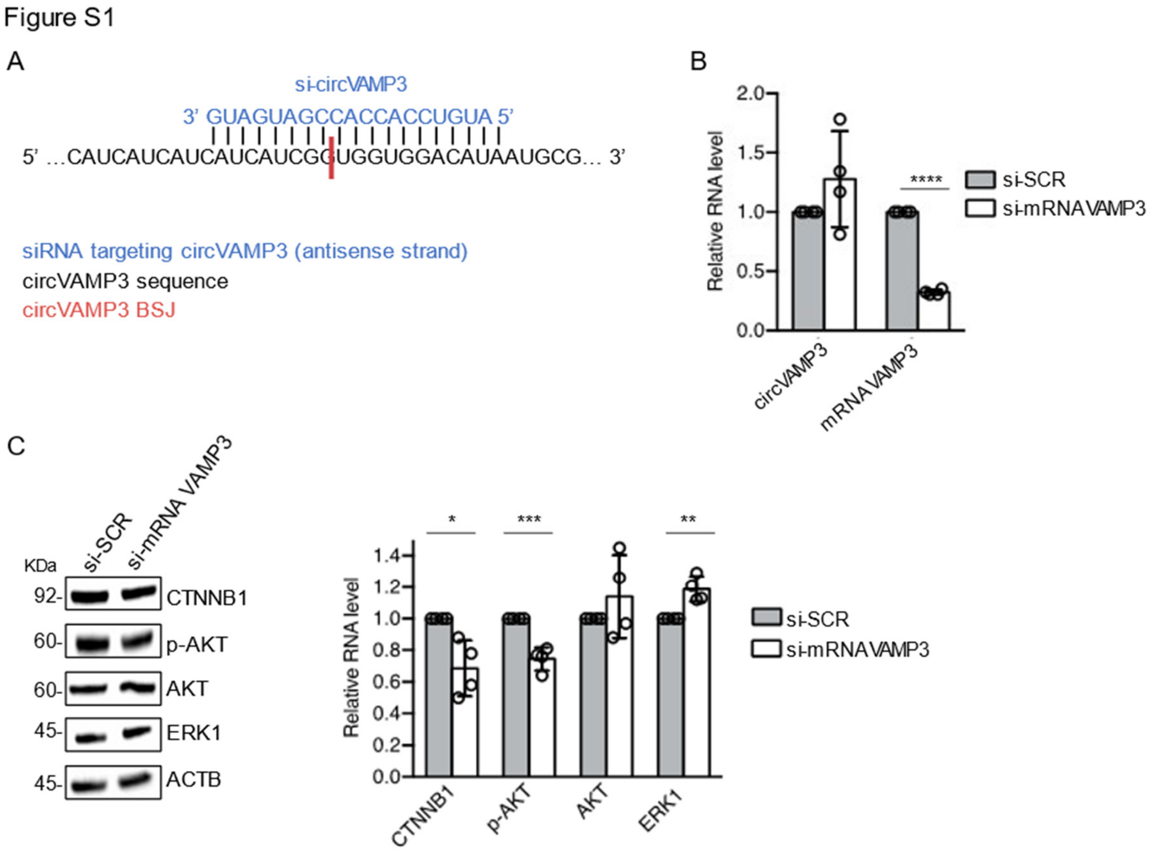

Figure S1. (**A**) Schematic representation of the BSJ region of circVAMP3 targeted by the siRNA (si-circVAMP3). (**B**) Relative levels of RNA detected by qRT-PCR of circVAMP3 and VAMP3 mRNA in RH4 cells treated with si-SCR or si-mRNA VAMP3. Data are represented as mean of fold changes ± standard deviation of 4 biological replicates, with dots representing individual datapoints. (**C**) Left panel: Western blot in RH4 cells treated either with si-SCR or si-mRNA VAMP3 to detect CTNNB1, phosphorylated-AKT (p-AKT), AKT, ERK1 and ACTB (loading control) protein levels. Protein molecular weights (KDa) are indicated on the left; the panel shows a representative experiment of four. Right panel: Quantification of protein levels (relative to ACTB) from the experiment shown in the left panel. Data are represented as mean of relative protein levels ± standard deviation of 4 biological replicates, with dots representing individual datapoints. Where statistical analysis was performed, the ratio of each sample versus its experimental control was tested by two-tailed unpaired Student's t test. *: p- value < 0.05, **: p-value < 0.01, ***: p-value < 0.001, ****: p-value < 0.0001.
